# A New River Sediment Standard Reference Material

**DOI:** 10.6028/jres.093.027

**Published:** 1988-06-01

**Authors:** Michael S. Epstein, Barry I. Diamondstone, Thomas E. Gills, John R. Adams

**Affiliations:** National Bureau of Standards, Gaithersburg, MD 20899; U.S. Army Corps of Engineers, Buffalo, NY 14207

Several sediment reference materials are available for use in assuring the quality of environmental measurements. These materials have been prepared by various organizations throughout the world, and include bottom sediments from numerous aqueous environments (rivers, ponds, bays, streams, gulfs, etc.) for which either certified or consensus concentration values of inorganic or organic constituents are or soon will be available [1]. As a result of the decreasing supply of Standard Reference Material (SRM) 1645, River Sediment, the NBS Office of Standard Reference Materials authorized the evaluation of candidate materials for a new river sediment SRM. Considerable emphasis was placed on assuring the homogeneity of the sample, the quality of the analyses, and the selection of certified constituents throughout the development of this material, SRM 2704.

Sample homogeneity and analysis quality are reflected in the uncertainty limits that are placed on the certified values. These limits can be used as a realistic estimate of the sum of error sources associated with the collection, processing, and certification of each element in the sample matrix. In round-robin type protocols involving inter- and intra-laboratory comparisons, it has been observed that within-method uncertainties are often significantly less than between-method uncertainties. Assuming that appropriate sampling protocols were followed and that the confidence limits reported for individual methods are not underestimated, it is reasonable to conclude that the imprecision and bias of individual techniques used by different laboratories produces a significant uncertainty in the approved value. As the constituent concentrations in these materials decrease, the uncertainty associated with reported values increases, since matrix interferences and contamination make larger contributions to the overall uncertainty of the individual analytical techniques. At the lowest concentrations, where the analyst begins to approach the detection limit of the technique, fundamental noise sources [2] become a major source of imprecision, and the number of useful techniques becomes limited.

Ideally, homogeneity information should provide an estimate of the amount of material required so as not to exceed a series of defined sampling uncertainties for each certified element. As discussed by Kratochvil and Taylor [3], homogeneity is estimated using the relationship *WR*^2^ = *K*_s_, where *W* is the weight of sample, *R* is the %RSD of sample composition, and *K*_s_ is Ingamell’s sampling constant, which is the weight of sample required to limit the sampling uncertainty to 1% with 68% confidence. The sampling constant can be determined by estimating the between-sample standard deviation from a series of measurement sets of different sample weights.

The planning that goes into the preparation of an SRM includes the selection of analytical techniques which have been shown to have adequate sensitivity and precision for specific analyses. In a laboratory such as NBS, which is dedicated to certification work, it usually is easier to select appropriate analytical methods that have been refined over the years, and to run these procedures by trained measurement experts, with readily implemented controls. The controls available in a dedicated certification laboratory usually result in much lower uncertainties than would be possible using a large number of cooperating laboratories in a round-robin consensus approach to certification. The goal of this project was to reduce the uncertainty associated with certified values for the new river sediment below those previously attained for SRM 1645.

Although the ideal sediment reference material should be certified for both inorganic and organic constituents, this is not always possible because the criteria for collection, processing and storage often are different for these two types of constituents. Often, the most desirable equipment for the collection of materials to be certified for organics are the most probable sources of contamination to an analyst interested in inorganics, and *vice versa*. The collection and processing procedures developed for SRM 2704 were designed to minimize inorganic contamination, but where possible, steps also were taken to keep organic contamination to a minimum.

Four grab samples of sediment were collected from the Buffalo, NY area in April of 1985. A series of tests were conducted to develop a scheme for producing a fairly homogeneous sample from a bulk sample, to evaluate various techniques for putting the sample in solution, and to determine the concentrations of the elements of interest. Based on the results of these tests, it was decided to sample from the bottom of the Buffalo River, in the vicinity of the Ohio Street bridge. The Buffalo River sediment was collected in cooperation with the U.S. Army Corps of Engineers in late November 1986, using an unpainted, cleaned, and rinsed clamshell bucket suspended from the crane of a derrick boat. It was known that the sampling site had not been dredged in over 2 years. A sample of proximately 12 to 24 inches of sediment was removed from each full bucket. The dredged sediment was transferred into 55 gallon steel drums lined with teflon bags nested inside of polyethylene bags. Teflon-coated shovels were used to transfer the material to the drums, and care was taken to ensure that the material transferred was not in direct contact with the inside of the bucket (fig. 1). Once the drums were filled, the bags and drums were sealed and transferred to a refrigerated truck. The entire collection was then transported to a facility where it was freeze-dried, screened to pass a 100-mesh sieve, and mixed in a stainless steel blender. The blended material was evaluated for gross homogeneity, and the bulk lot was radiation sterilized at a minimum dose of 2.5 megarads.

The relative homogeneity of the material was assessed using analysis of variance (ANOVA) to separate bottle-to-bottle, sample-to-sample, and instrument variance. Instrument measurement precision was degraded to approximately 1–2% relative by the high dissolved solids content of the samples. No inhomogeneity for Na or Si could be detected when compared to the instrumental measurement variance. Inhomogeneity was definitely observed for the Cr, and marginally for the Fe. Based on this information, the material was reblended. The material was then bottled in 50 g units, producing a total of 3305 units. Fifty bottles, randomly selected from the entire population, were used for the analytical measurements.

The corroborating laboratories and the techniques selected for certification analysis are listed in table 1. Each laboratory was given a set of instructions which detailed the number of samples to be analyzed, the dissolution procedures, the control samples to be analyzed, and the information relative to method bias and precision which should be included with the individual reports of analysis. It is expected that all results will be received by the end of 1987, and the new SRM should be available by the spring of 1988.

## Figures and Tables

**Figure 1:**
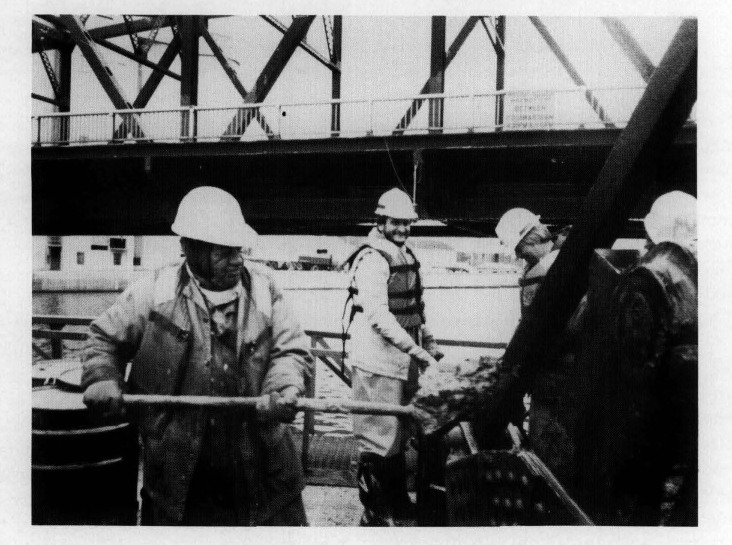
River sediment being transferred from collection bucket to teflon-lined barrels using teflon-coated shovels.

**Table 1:** Corroborating laboratories and techniques selected for certification analysis

*Corroborating Laboratories*
National Research Council of Canada, Analytical Chemistry Divison
Los Alamos National Laboratory
Oak Ridge National Laboratory
Penn State University, Mineral Constitution Laboratory
Shanghai Institute of Testing Technology, Peoples Republic of China
Virginia Institute for Marine Science
*Analytical Techniques*
Inductively Coupled Plasma Spectroscopy (ICP)
Instrumental Neutron Activation Analysis (INAA)
Inert Gas Fusion (IGF)
Cold Vapor Atomic Absorption Spectroscopy (CVAAS) Polarography
Isotope Dilution Mass Spectrometry (IDMS)
Gravimetry
Inductively Coupled Plasma—Mass Spectrometry (ICP-MS)
Laser Enhanced Ionization Spectroscopy (LEI)
Potentiometry
Direct Current Plasma Spectroscopy (DCP)
Ion Chromatography (IC)
Coulometry
Photometry
Hydride Generation Atomic Absorption Spectroscopy (HGAAS)
